# Supplementary material for: Oleanolic Acid Promotes the Formation of Probiotic Escherichia coli Nissle 1917 (EcN) Biofilm by Inhibiting Bacterial Motility
Source: Microorganisms. 2024 May 29;12(6):1097. doi: 10.3390/microorganisms12061097 (PMC11205495; doi:10.3390/microorganisms12061097)
Supplement: Supplementary file 1 [file microorganisms-12-01097-s001.zip › microorganisms-2987478-supplementary.pdf]

Table S1. Gene primer sequence

Gene Name	Upstream primers (F)	Downstream primers (R)
16SrRNA	TGCGTAGAGATCTGGAGGAATAC	ACCAGGGTATCTAATCCTGTTTG
lpp	AATCCTGGGTTCTACTCTGCTG	TTGCTCAGCTGGTCAACTTTAG
cpxp	AAATTTTGATGAAAACGCTGTG	TAACAGGCGATACATTTGGTTG
FliA	GAACGCTATGACGCCCTACA	TCCAGTTGCCCTATTGCCTG
slp	CGCAAAATATCAAAGGCAATAAC	GTTGATAACCTTCCCACCAAAG
ybtP	GATAGCCAATAACATTCGTCTGG	CGACTCGTGTGTTGTAACCTTC
ybtQ	CAAAACTACGGTCACCAAACTG	ACAGAAATCAGGCTATTGAGCTG
fimA	TTGTTCTGTCGGCTCTGTCC	ACTGGTTGCTCCTTCCTGTG

Table S2. Transcriptome sequencing data results statistics

Sample Name	Reads No.	Bases (bp)	Q30 (bp)	N (%)	Q20 (%)	Q30 (%)
CONT-1	3147 7224	4753060824	4562150770	0	97.8	95.98
CONT-2	38 199088	5768062288	5537657778	0	97.81	96.01
CONT-3	36370434	549 1935534	5266632289	0	97.77	95.9
OA-1	35737 754	5396400854	51 79082317	0	97. .81	95.97
OA-2	38070862	5748700162	5516198918	0	97.78	95.96
OA-3	35329162	5334703462	5117308838	0	97.78	95.92

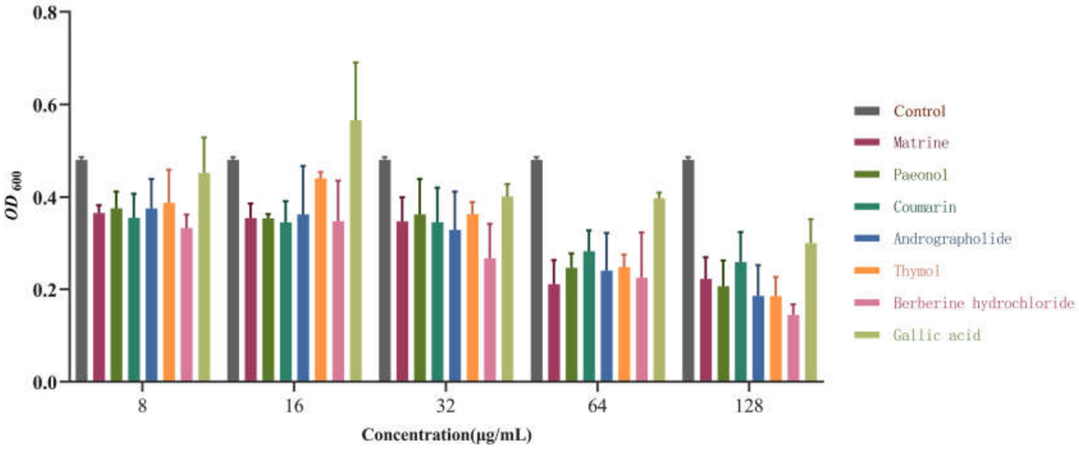

Figure S1. The influence of other candidate Chinese medicine active substance on the formation of EcN biofilms
